# Neutral Red-carbon nanodots for selective fluorescent DNA sensing

**DOI:** 10.1007/s00216-022-03980-1

**Published:** 2022-03-14

**Authors:** Emiliano Martínez-Periñán, Álvaro Martínez-Sobrino, Iria Bravo, Tania García-Mendiola, Eva Mateo-Martí, Félix Pariente, Encarnación Lorenzo

**Affiliations:** 1 Departamento de Química Analítica y Análisis Instrumental, Universidad Autónoma de Madrid, 28049 Madrid, Spain; 2 Institute for Advanced Research in Chemical Sciences (IAdChem), Universidad Autónoma de Madrid, 28049 Madrid, Spain; 3 Centro de Astrobiología (CSIC-INTA), Ctra. Ajalvir, Km. 4, Torrejón de Ardoz, 28850 Madrid, Spain; 4 IMDEA-Nanociencia, Ciudad Universitaria de Cantoblanco, 28049 Madrid, Spain

**Keywords:** Carbon nanodots, Neutral Red, *Escherichia coli*, Fluorescence DNA assay

## Abstract

Carbon nanodots modified with Neutral Red covalently inserted in the nanostructure (NR-CDs) have been prepared by a simple synthesis method based on microwave irradiation under controlled temperature and pressure. The synthetized NR-CDs have been characterized by different techniques, demonstrating the covalent bonding of Neutral Red molecules to the carbon dots nanostructure. Fluorescence activity of the prepare NR-CDs has been explored showing different interaction pathways with singled and doubled stranded DNA. These studies have been successfully applied to develop a new fluorescence DNA hybridization assay to the detection of a specific DNA sequence of *Escherichia coli* bacteria.

**Graphical abstract:**

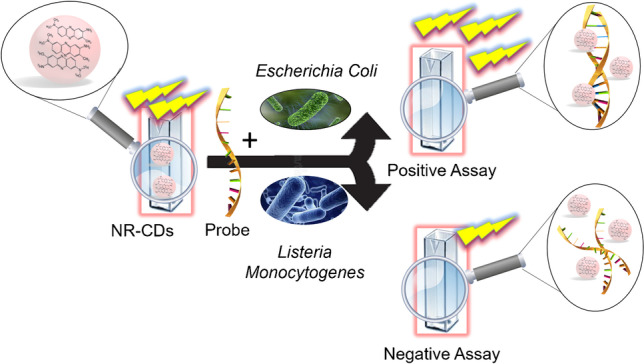

**Supplementary Information:**

The online version contains supplementary material available at 10.1007/s00216-022-03980-1.

## Introduction

Carbon nanomaterials have been widely used during the last decades in multitude of applications and fields as biomedicine [1], electronic industry [2], environmental applications [3], energy [4], sensors [5], and biosensors [6]. Despite of the interesting properties and capabilities discover for the different carbon nanomaterials, there is a great trend that tries to manipulate their interesting properties according to our needs. Therefore, there are multitudes of research projects aimed at carbon nanomaterials functionalization to endow them with interesting properties [7–10]. In this sense, new synthetic methods, especially those based on bottom-up strategies, allow a fine control of synthetic procedures, opening new possibilities of functionalization and insertion of specific moieties in nanomaterial structure as have been described in graphene nanoribbons [11] and nanocomposites material [12].

Among carbon nanomaterials, carbon nanodots (CDs) are the allotropic specie of carbon last discovered [13]. A great interest about CDs, regarding their extraordinary properties (water solubility, low cytotoxicity, high photoluminescence, simplicity of their synthesis), has emerged in recent years [14]. Their easy synthesis is usually based on solvothermal methods, using as precursor compounds very abundant in the nature as saccharides, amino acids, and organic acids. Within different synthetic protocols for producing CDs, the bottom-up approach has shown that the choice of the starting materials is critical. For example, choosing the proper precursors, the presence of heteroatoms can be modulated in order to obtain in situ doped CDs with specific properties [15–17]. Furthermore, new synthetic protocols are using specific organic moieties as precursor of CDs with the aim of providing certain reactivity or specificity to the CDs nanostructure. This is the case of the use of some quinones [18] to customize the electrochemical behavior of CDs, and the use of porphyrin‐containing carbon dots [19], used to generate cytotoxic singlet oxygen upon irradiation, and induce cell apoptosis.

Following this research line, herein, we report a simple synthetic strategy based on fast and controlled microwave heating, to obtain the in situ tuning of CDs optical properties by introducing Neutral Red (NR) in their structure. Phenazines present antimicrobial activity, anti-oomycete activity, genotoxicity, and cytotoxicity [20]. Based on their unique electrochemical and optical properties, they have also different important applications such as in the development of electrochemical sensors [21] and biosensors [22], optical assays [23], in particular those based on their fluorescence [24, 25], and fabrication of dye-sensitized solar cells (DSSCs) [26–28]. All of these excellent properties can be acquired by a nanomaterial as CDs, if it is modified with a phenazine. It has been previously described the modification of CDs with NR by supramolecular interaction in order to combine their specific properties at analytical applications [29, 30]. Neutral Red molecules have also covalent linked to carbon dots in a post-synthetic step to be used as an electrochemical redox probe [31]. Recently, carbon dots modified with Neutral Red have been obtained by hydrothermal methods using the phenazine as precursor together with thiourea [32, 33] or citric acid [34]. Hydrothermal methods usually require long synthesis times. Their specific spectroscopic and electrochemical properties have been applied as analytical tools, demonstrating the great advantages of combining carbon nanostructures and the phenazine.

Simple and sensitive sequence-specific DNA detection is fundamental not only in the area of clinical molecular diagnostics of diseases and biomedical studies but also in microbiology, food control and security, and environmental monitoring. As a powerful analytical tool, fluorescence-based systems have been widely employed for DNA sequences detection due to the advantages of them including sensitivity, specificity, and cost-effectiveness. In particular, recognition of specific DNA sequence from a pathogen as a fast and sensitive detection method is arising, since pathogenic bacteria and viruses are responsible of serious health diseases [35]. It is well known the health problems caused by the contamination of food or water with the bacteria *Escherichia coli*. Hence, this research field is open to new assay developments and advanced functional materials, such as carbon nanomaterials, that have given into novel pathway for the establishment of fluorometric sensing strategies [36]. In fact, CDs have been successfully applied in the development of a fluorometric DNA method of detecting DNA hybridization [37], being used even for the rapid detection of gene mutations. However, one of the drawbacks of these assays is that CDs need excitations wavelengths in the range of UV. Although much effort has been put into improving their optical performance, there is still much room for improvement. In this sense, the modification of CDs with a molecular specie capable of being excited in the visible wavelength range will entail an advantage, by allowing the use of affordable equipment and the direct application in well plates.

In this work, we report a new assay strategy for fast detection of specific DNA sequences of *E. coli* based on a functional carbon material: Neutral Red-carbon nanodots (NR-CDs) synthesized using, for the first time, a microwave assisted reactor, which allows the complete synthesis process of NR-CDs in only 3 min. It is worth to note that the developed procedure does not require a post-modification of CDs after their synthesis to include the NR molecule.

## Materials and methods

### Chemicals

Sodium chloride, dibasic and monobasic sodium phosphate, L-arginine, 3,3′-diamino-N-methyldipropylamine, NR chloride, double-stranded calf thymus DNA (dsDNA), DNA oligonucleotides, and all other chemicals used in this work were purchased from Merck. All solutions were prepared using water purified with a Millipore Milli-Q-System (18.2 MΩ cm). Dialysis membrane tubing cutoff in the range of 0.1–0.5 kDa was provided by Spectrum Laboratories.

### DNA samples

Double-stranded DNA from calf thymus (dsDNA) and 25-base oligonucleotides-specific sequences from *Escherichia coli*, *Salmonella enterica*, and *Listeria monocytogenes* bacteria used in this work were purchased from Merck and are listed in Table 1. The DNA sequences used comprise the DNA probe (a single-stranded sequence complementary to the analyte named as *E. coli* probe), the target sequence (a specific sequence from *Escherichia coli* bacteria, denoted as *E. coli*), and two non-complementary sequence (a specific sequence from other bacteria, *Salmonella Enterica* and *Listeria Monocytogenes*, denoted in the text as *Salmonella* and *Listeria*, *r*espectively) used as potential interferents.

**Table 1:** DNA sequences used

Oligonucleotides sequences		
Probe	5′-CAGGATATGTGGCGGATGAGCGGCA	*E. coli* probe
Complementary	5′-TGCCGCTCATCCGCCACATATCCTG	*E. coli*
Non-complementary (Interference)	5′-GTACGCTTCGCCGTTCGCGCGCGGC	*Salmonella*
Non-complementary (Interference)	5′-AGTGAGTGCGGTTAGACCTGCTAGG	*Listeria*

### Real bacteria culture samples

Genomic DNA was extracted from the cultured *Escherichia coli* (4.31·10^6^ CFU/mL; 215.7 ng/µL). Firstly, 50 µL of *Escherichia coli* bacterial strains (positive sample) or *Lactobacillus rhamnosus* (6.23·10^6^ CFU/mL; 225.2 ng/µL) (negative sample) were streaked on brain heart infusion (BHI) with 25% glycerol and incubated at 37.0 °C for 24 h. Then, 1 mL of each bacterial culture suspension was used for DNA extraction using the E.Z.N.A Bacterial DNA kit (Omega Bio-TEK) according to the manufacturer’s instructions. A unique type of cell colony with a positive growth was obtained in purity test.

*Escherichia coli* and *Lactobacillus rhamnosus* genomic DNA samples were denatured by boiling in water capped vials containing the samples for 30 min followed by rapid cooled. To prevent spontaneous re-naturalization, this solution was subsequently quenched in an ice-bath. Finally, 50 µL of denatured sample were diluted to a final volume of 200 µL using 50 µL of 10 mM phosphate buffer (pH 6.8) containing 800 nM of *E. coli* probe and 100 µL of 5.6 µg/mL NR-CDs.

### Apparatus

A Cary Eclipse Varian fluorescence spectrophotometer equipped with Cary Eclipse Microplate Reader and a Thermo Scientific™ Multiskan™ GO spectrophotometer were used for fluorometric and spectrophotometric measurements, respectively. 96-well microplates were used for absorbance titrations and were supplied by JET-BIOFIL. 96-well microplates for fluorescence-based assays (Microfluor®2white Thermo Scientific) were used for fluorescence titrations and *E. coli* determination fluorescence assays.

A CEM Discover microwave system (Matthews (NC), USA) was used for NR-CDs synthesis.

Fourier transform infrared (FT-IR) spectra were recorded from KBr pressed pellets of the solid material and precursors in the wavelength range 5000–500 cm^−1^ using a Brucker IFS60v spectrometer.

For transmission electron microscopy (TEM), Lacey carbon support film copper grids (400 mesh, Electron Microscopy Sciences) were used. Images were obtained with a JEOL JEM 2100 electron microscope.

X-ray photoelectron spectroscopy (XPS) analysis of the samples was carried out with a Phoibos 150 MCD spectrometer equipped with hemispherical electron analyzer, and using an Al Ka X-ray source (1486.7 eV) with an aperture of 7 mm × 20 mm. The base pressure in the ultra-high vacuum chamber was 2 × 10^−9^ mbar, and the experiments were carried out at room temperature. A 30 eV pass energy was applied for taking the overview sample, whereas 20 eV pass energy was applied for the analysis of the following core level spectra: O (1 s), C (1 s), and N (1 s). XPS spectra regions were fitted and deconvoluted using the fitt-xps software, and calibration was done against the Au (4f _7/2_) peak set to 84.0 eV for the gold surface sample. For XPS, gold plates (12 mm × 12 mm, Arrandee TM Supplies, Germany) were modified by drop casting with suspension of the nanomaterial and letting them dry.

### Procedures

#### NR-CDs synthesis

NR-CDs were synthesized following the next procedure. In a typical synthesis, 87 µg L-arginine, 72 µg Neutral Red chloride, 86 µL 3,3’-diamino-N-methyldipropylamine, and 100 µL Milli-Q water were irradiated in a microwave system at a constant temperature of 235 °C and a maximum pressure of 20 bar during 180 s. Then, the red solid obtained was dissolved in 10 mL of Milli-Q water and filtered using 0.1 µm porous filter. The suspension was dialyzed in a 0.1–0.5 kDa dialysis membrane for 10 days. The concentration of as-prepared NR-CDs was 1.09 mg/mL. The resulting solution was stored at 4 °C. A fraction of the NR-CDs suspension was freeze-dried to carry out FT-IR and XPS experiments. To compare the effective modification of carbon nanodots with NR, carbon nanodots without NR were synthesized following the same procedure without the addition of NR, as it is reported in the literature [38]. In order to probe the covalently linkage among NR and CDs structure during NR-CDs synthesis process, a control experiment mixing NR and CDs in Milli-Q water, keeping the mixture at room conditions, was carried out (CDs + NR). The objective of this experiment is analyzing the contribution of NR adsorption over the CDs surface without the generation of new covalent bonds. A fraction of the NR + CDs suspension was freeze-dried to carry out FT-IR and XPS experiments.

#### DNA solution preparation and denaturation

dsDNA stock solutions (1.0 mg/mL) were prepared in Milli-Q water. The concentration in base pairs (bp) of DNA was determined by using the molar absorptivity (6600 L/(mol cm)) of DNA at 260 nm. Single-stranded calf thymus DNA (ssDNA) was obtained by boiling in water the vials containing dsDNA for 30 min, cooling them in an ice-bath, and stored frozen at − 20 °C.

*E. coli* solutions: probe, complementary sequence (*E. coli*) and interference sequences (*Salmonella* and *Listeria*) were prepared from the 100 µM stock using 10 mM phosphate buffer (PB) pH 7.0 as solvent. These solutions were stored at − 20 °C.

#### Interaction of DNA and NR-CDs

Absorbance and fluorescence titrations of NR-CDs and calf thymus DNA were carried out fixing NR-CDs concentration and varying the concentration of dsDNA and ssDNA from 1.0 to 150.0 µM. In all cases, 0.1 M PB 7.0 pH were used as a solvent. The mixture solutions were allowed to react for 30 min at 23 °C before fluorescence spectra were monitored using an exciting wavelength of 530 nm directly at the well-plates.

#### Calculation of the intrinsic binding constant between NR-CDs and dsDNA, Kb

The intrinsic binding constants, *Kb*, were calculated by using the data from absorption titration and the equation previously reported by Meehan et al. [39]:

1 $$\left[\mathrm{ADN}\right]/\left(\varepsilon a-\varepsilon f\right)=\left[\mathrm{ADN}\right]/\left(\varepsilon b-\varepsilon f\right)+1/Kb\left(\varepsilon a-\varepsilon f\right)$$

where *εa* is the molar absorptivity of NR-CDs in the presence of the different concentrations of dsDNA, and *εf* and *εb* are the molar absorptivity for free and bound forms of NR-CDs, respectively.

From the plot of [*ADN*] / (*εa* − *εf*) versus (*εa* − *εf*), the value of 1/*Kb* was obtained.

#### *E. coli* determination by fluorescence assays

Different concentrations of complementary (*E. coli*) or non-complementary sequence (*Salmonella* and *Listeria*) were incubated with *E. coli* probe (200 nM) for 2 h at 40 °C in 0.1 M pH 6.8 phosphate buffer, in order to allow hybridization. Then, NR-CDs were added in the wells, fixing the NR-CDs concentration at 2.8 µg/mL. Solutions were let to react for 30 min at 23 °C before fluorescence was monitored at 590 nm with an excitation wavelength of 530 nm at room temperature, directly in the microwell plate.

## Results and discussion

### Synthesis and characterization of NR-CDs

NR-CDs were synthesized in a microwave reactor, using Neutral Red chloride, L-arginine, and 3,3′-diamino-N-methyldipropylamine as precursors as described in detail in the experimental section and following a similar procedure described by Arcudi et al. [15, 18] with some variations.

**Fig. 1:**
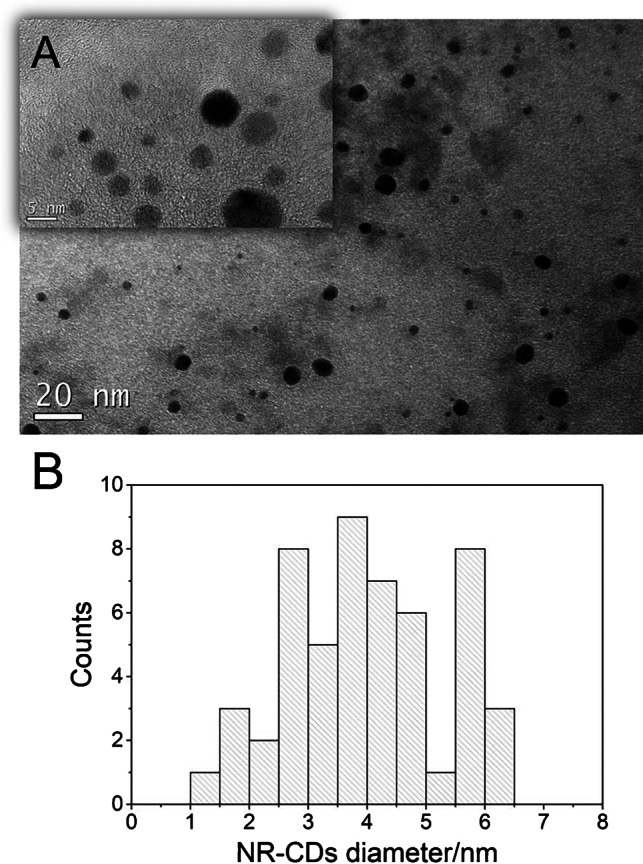
TEM images of NR-CDs **A** and **B** diameter histogram obtained by TEM

NR-CDs have been exhaustively characterized. The TEM image (Fig. 1A) shows NR-CDs morphology as quasi-spherical nanoparticles. The size distribution of NR-CDs obtained by measuring the average size of around 70 CDs (see histogram at Fig. 1B) indicates an average size of 4.7 nm ranging from 1.0 to 6.5 nm in diameter.

**Fig. 2:**
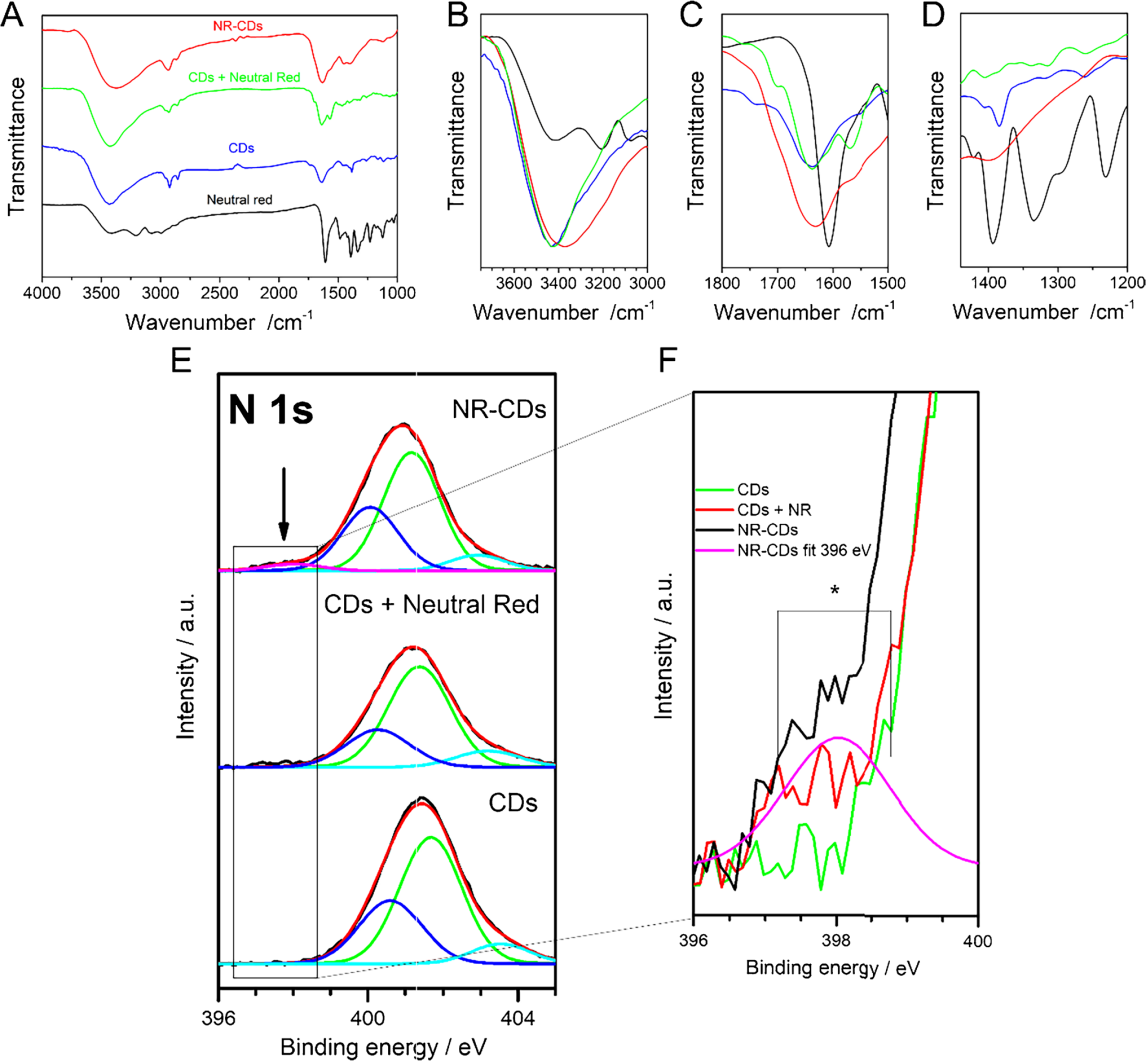
**A** FT-IR spectra of NR-CDs (red), CDs (blue), CDs + NR (green), and NR (black). Amplification of FT-IR spectra of Fig.2A in the range 3750–3000 cm^−1^ **B**, 1800–1500 cm^−1^ **C**, and 1450–1200 cm^−1^ **D**. **E** XPS photoemission spectra of N 1 s core level of NR-CDs, CDs + NR, and CDs drop casted on gold surfaces and **F** XPS photoemission spectra amplification between 396 and 400 eV

Figure 2A shows the FT-IR spectrum of NR-CDs, CDs + NR, CDs, and NR. As can be observed in the NR spectrum, a band at 3336 cm^−1^ related to primary amine (N–H stretch asym) and a band at 3147 cm^−1^ caused by the (N–H stretch asym) appear. The band around 1617 cm^−1^ is a consequence of the N–H bending. The FT-IR of CDs shows the stretching band of OH and NH_2_ as a broad band centered at 3436 cm^−1^, the bands around 2930 cm^−1^ derived from the C-H bond stretching vibration. C = O stretching vibrations appear at 1644 cm^−1^. At 1562 cm^−1^ appears a band ascribed to the C = N stretching, while the bands at 1457 cm^−1^ and 1384 cm^−1^ are related with C-N bonds. The 1071 cm^−1^ bands correspond to the alcoxy (C-O) stretching vibrations. FT-IR spectrum of NR-CDs shows similar features to that of CDs. However, slight differences are observed and point to modification of CDs with the phenazine (NR). In particular, the wider band present at the NR spectra shows a shift from the 3431 in CDs to 3360 in NR-CDs (Fig. 2B). At the same time, the intensity increase of the band at 1633 cm^−1^ in the NR-CDs spectrum is also associated with NR (Fig. 2C). Furthermore, the presence of a wide band at 1440–1358 cm^−1^ is also related to the NR spectra (Fig. 2D). The appearance of these new IR features after NR interaction on the CDs (NR-CDs), although they are not conclusive results, seems to indicate compositional changes that we attribute to the presence of NR, therefore pointing out the successful NR insertion in the CDs structure. Furthermore, the appearance of these significant IR features is less remarkable for the CDs + NR (green spectra) case, meaning a significance difference between both interaction processes: adsorption in the case of CDs + NRs or covalent linkage of NR into the CDs nanostructure in the case of NR-CDs.

In order to confirm the successful NR insertion in the CDs structure, complementary information about chemical identity of the NR-CDs, CDs + NR, and CDs drop casted over Au plate surfaces were obtained by XPS analysis. Figure 2E shows the N 1 s core level peaks of the CDs, CDs + NR, and NR-CDs (covalent linkage) samples. High-resolution spectra of nitrogen show complex features, which were carefully decomposed. A best-fit of the N 1 s core level shows three contributions at 400.6–400.1 eV, 401.2–401.6 eV, and 403.0–403.5 eV which are assigned to nitrogen in N-(C3), C = N (cycles), and N = positive charges (quaternary) species, respectively [40]. In addition, the NR-CDs compound presents an extra forth nitrogen component at 398.0 eV (pink line) and Fig. 2F, which would be assigned to the pyridinic nitrogen (imine) structure [40–42], that is ascribe to nitrogen with only one electron pair, located either at the edge of the graphitic network or next to a vacancy, and bonded to two carbon atoms. The appearance of the new component only for the NR-CDs case suggests the chemical structure modification due to the interaction between the colorant NR and the CDs compound, which does not occur for the CDs + NR case. Therefore, the XPS data confirm the covalently insertion of NR compound on the NR-CDs case, whereas for the CDs + NR does not take place. Additionally, the C 1 s peak for the NR-CDs shows noteworthy changes in its shape respect to the CDs and CDs + NR cases (see Fig. SI 1). Even if the C 1 s core level peak presents four similar components at 285.0 eV, 286.7 eV, 288.0 eV, and 289.7 eV for all cases, the ratio between the components changes for the NR-CDs sample. The two first components increase their intensity, and the two last components decrease, which causes a significant change in the profile shape carbon spectra, whereas the CDs and CDs + NR cases show a very similar peak profile in both cases for the carbon region. These results are in good agreement with the previously described chemical modification for the NR-CDs case.

FT-IR and XPS complementary techniques prove a remarkable difference between the studied cases, first confirming the interaction between the colorant NR and the CDs in both cases CDs + NR and NR-CDs, and second the effective covalently insertion only in the NR-CDs case.

The *Z*-potential of NR-CDs in water was determined to be + 17.8 mV, which suggests a positive charge on the surface in agreement with the presence of protonated amines functional groups. However, the zeta potential of synthesized CDs (without NR) was − 10.3 mV. This negative value agrees well with that determined for other CDs previously synthetized by a similar method [38]. The change from a negative to a positive value of zeta potential for CDs and NR-CDs, respectively, confirms the compositional change that takes place by the NR-CDs formation demonstrated by XPS and FT-IR. A positive zeta potential value is indicative of an increase of pyridinic nitrogen (imine) structures, which are usually protonated in solutions of pH below 7, as in the present case (pH around 5).

**Fig. 3:**
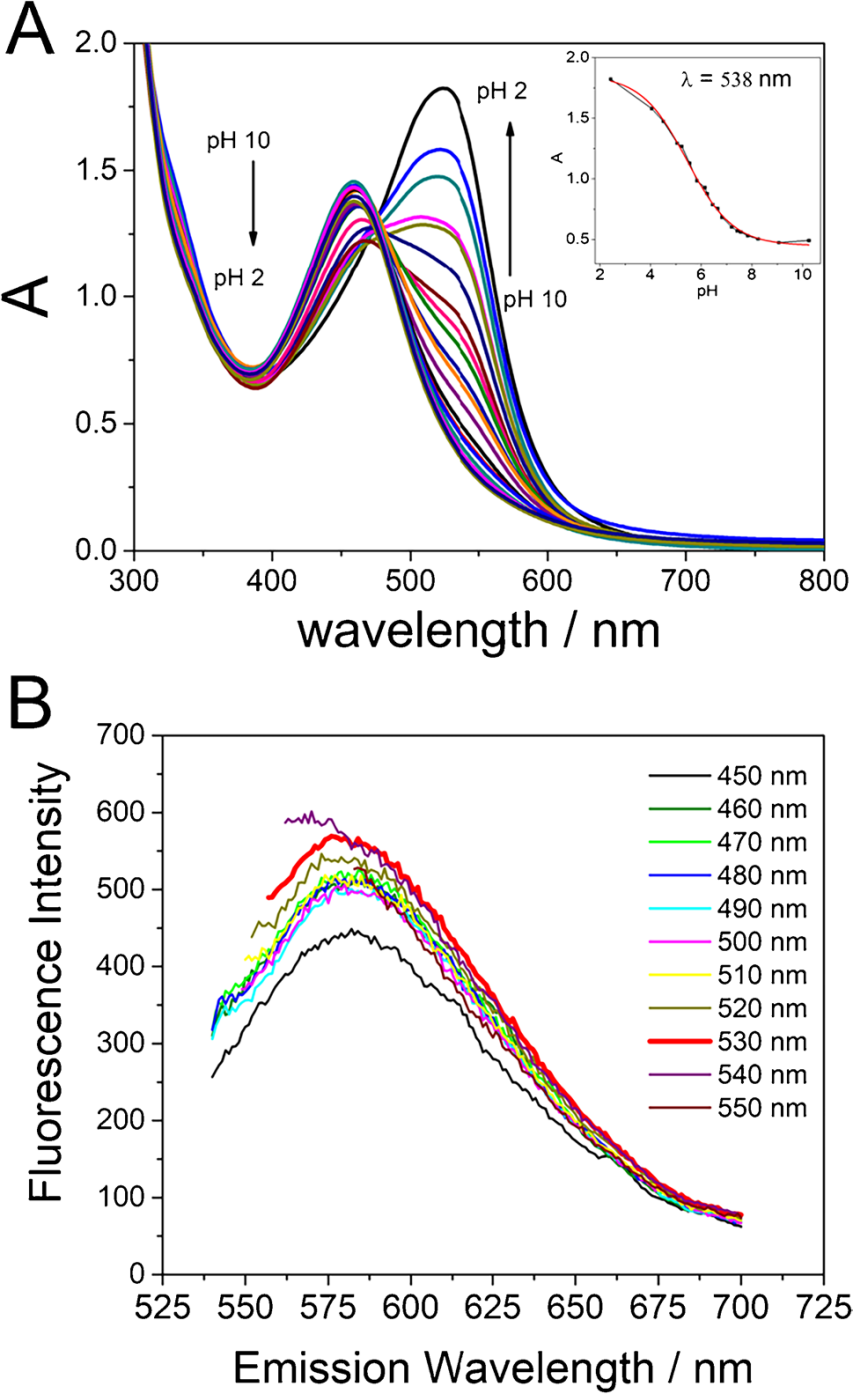
**A** UV–Vis spectrophotometric spectra of 50.0 µg/mL of NR-CDs at different pH values. Inset: absorbance value measure at *λ* = 538 nm at different pH values. **B** Fluorescence spectra at different excitation wavelengths of 21.8 µg/mL of NR-CDs at pH 7.0

In aqueous solution, NR can be protonated or not depending on pH solution according to its pK_a_ (around 6.50). Both forms show a single absorption band, at 452 nm for the non-protonated and at 528 nm for the protonated form (data not shown). Hence, in order to assess if the synthetized NR-CDs present the characteristic absorption bands of NR, we recorded the absorbance spectra of a suspension containing NR-CDs (21.8 µg/mL) at pH from 5 to 9 (Fig. 3A). At pH 9, the expected band at 460 nm ascribed to the non-protonated form of NR molecule is observed. At pH 5, in addition to the band at 538 nm due to the protonated form, we can observe a huge shoulder around 460 nm, which indicates a clear equilibrium between the protonated and non-protonated form of NR molecules inserted in the nanostructure. Same bands are observed at pH 7, in this case the protonation equilibrium has shifted to the non-protonated form decreasing the band at 538 nm, which is observed as a shoulder of the main band at 460 nm. This behavior is quite similar to the once described for NR molecules in solution, but the pK_a_ of NR-CDs has been estimated as 5.9 (Fig. 3A-Inset). This value is quite different to the pK_a_ of NR molecules. This fact together with shift of the UV–Vis absorption band maximum is due to the new covalent bonds formed among the NR molecules inserted in the carbon dots nanostructure, which varies its electronic structure.

Fluorescence spectra have also been studied using the colorant NR visible absorption wavelength to excite the NR inserted molecules, generating a fluorescence emission in the visible spectra region. As can be observed in Fig. 3B, unless the predominant specie at pH 7.0 is the non-protonated (maximum absorption band at 460 nm), the fluorescence emission increases when 530 nm wavelength is used to excite, pointing out the higher emission of the protonated form of the NR molecule.

### NR-CDs and DNA interaction

The interaction of DNA with molecules can affect their electronic structure, what generates changes in their absorption spectra [43]. With the aim of studying the interaction of the developed nanomaterial (NR-CDs) with DNA, we carried out a titration of NR-CDs with calf thymus DNA by increasing the amount of DNA and keeping constant the NR-CDs concentration. dsDNA and ssDNA have been used in order to prove if there is any difference in the mode of interaction with them. Fig. SI 2 shows the absorption spectra of NR-CDs without and with increasing concentrations of ssDNA (A) and dsDNA (B). In both cases, an absorbance decrease (hypochromic effect) of the band at 460 nm is observed, as well as a slight shift towards higher wavelengths (bathochromic effect). Both effects are more evident in the case of dsDNA. These changes in the absorption spectra of NR-CDs caused by DNA are a consequence of the local polarity changes around the chromophore due to strong interaction with pyrimidine and purine bases [44]. From the absorption titration data, it can be calculated the intrinsic binding constant, Kb, as described in detail in experimental section. This constant gives a quantitative estimation of the interaction strength. The calculated *Kb* values were (2.5 ± 0.5) ·10^5^ and (5.0 ± 0.3) ·10^4^ M^−1^ for dsDNA and ssDNA, respectively. This result suggests a stronger interaction with dsDNA than with ssDNA. Fluorescence measurements of the ssDNA and dsDNA titration (Fig. S12C and D, respectively) show that an ssDNA concentration increase leads to a decrease of NR-CDs fluorescence emission, while an increment of dsDNA concentration is associated with the opposite effect, an increase of the fluorescence emission. The fluorescence of the protonated NR-CDs species enhances when interact with dsDNA, while the opposite effect happens when protonated NR-CDs interact with ssDNA due to the different interaction mechanisms among them. This effect is the starting point of the use of this nanomaterial (NR-CDs) as an indicator of DNA hybridization. Therefore, hybridization fluorescence assays can be carried out using NR-CDs in solution, choosing the proper DNA probe sequence.

### Fluorescent assay for rapid *Escherichia coli* detection

Based on the results obtained with calf thymus DNA, we decided to go a step forward and to apply NR-CDs for the development of a fluorescence assay for the detection of a 25-mer specific sequence from the bacteria *E. coli*, using them as a hybridization event detector. The assay is based on the changes in the fluorescence of NR-CDs after interaction with the DNA capture probe (a 25-mer synthetic sequence from *E. coli*) before and after hybridization with the target (a fully complementary sequence to the probe or non-complementary sequence used as control) under optimal conditions of pH, concentration of NR-CDs, and temperature.

To optimize the pH of the assay, different pHs from 6.0 to 8.0 at a concentration of the recognition sequence (*E. coli* probe) of 200 nM were assayed. The fluorescence emissions before and after addition of 200 nM of *E. coli* sequence were recorded for each pH. The difference between signals due to the probe before and after hybridization with the target analyte was higher at pH 6.8 (see Fig. SI 3A). The concentration of NR-CDs was also optimized. Best results were obtained for 2.8 µg/mL of NR-CDs at 200 nM of *E. coli* probe (Fig. SI 3B). Poor signals were obtained when concentrations under 2.8 µg/mL of NR-CDs were employed.

**Fig. 4:**
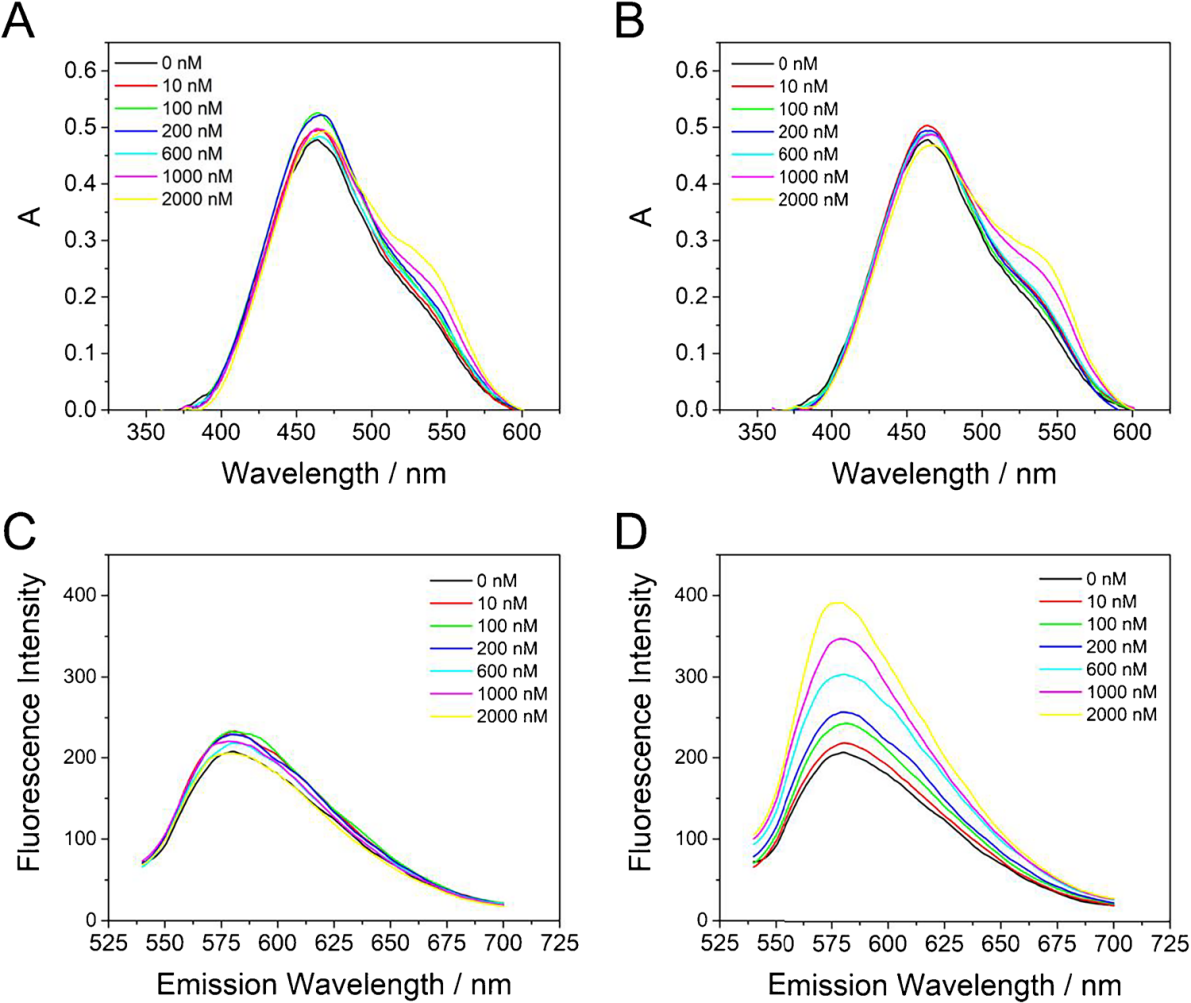
UV–Vis absorption (**A**, **B**) and fluorescence (**C**, **D**) spectra of NR-CDs in 0.1 M PB pH 7 in the absence and in the presence of increasing concentrations (0–2000 nM) of ss (**A**, **C**) and ds (**B**, **D**) DNA from *E. coli*

**Fig. 5:**
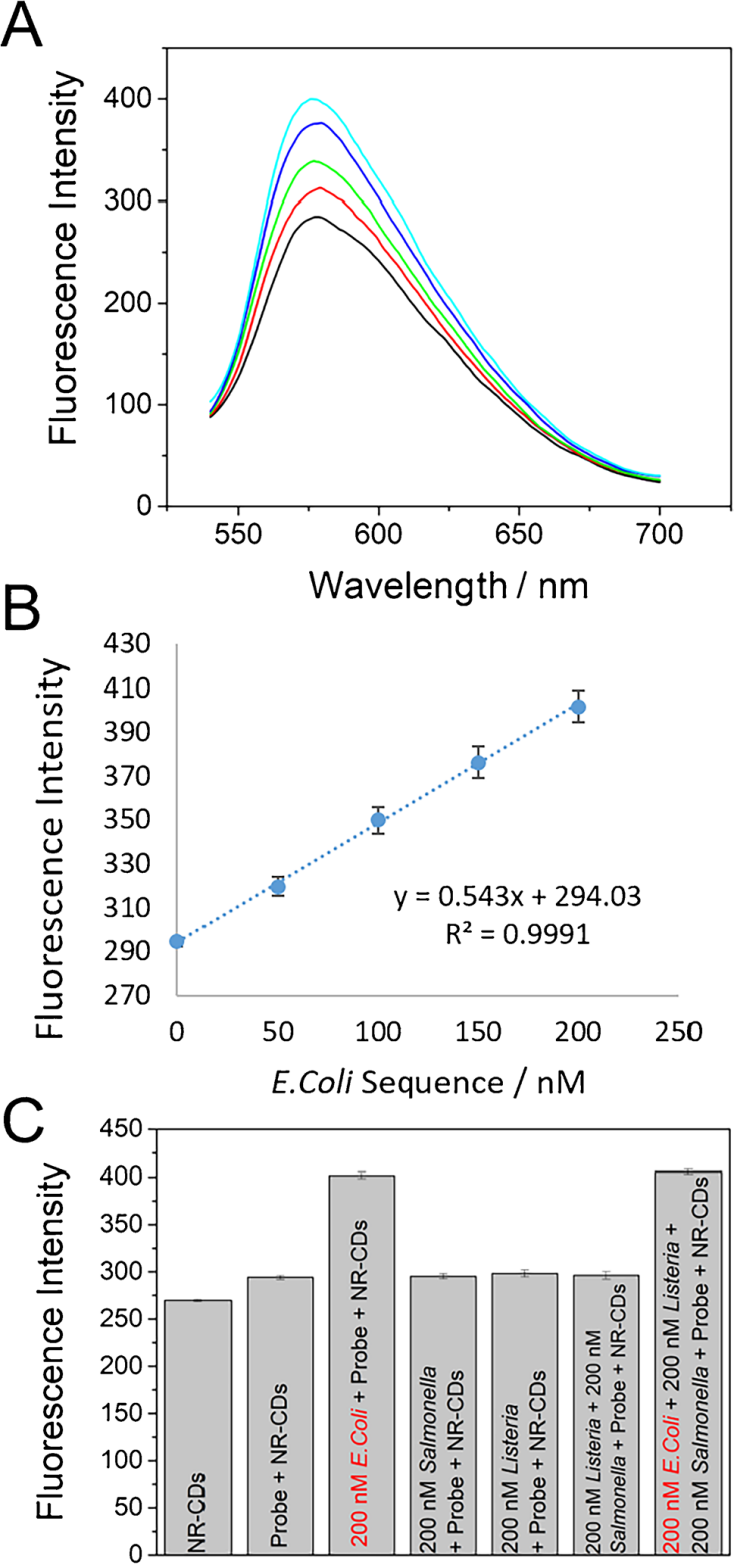
**A** Fluorescence spectra of NR-CDs (2.8 µg/mL) in the presence of *E. coli* probe before (black) and after the hybridization with increasing amounts of *E. coli* sequence (50 nM red, 100 nM green, 150 nM blue, and 200 nM cyan) using *λ*_exc_ = 530 nm. **B** Calibration plot obtained. **C** Fluorescence intensity obtained of NR-CDs, NR-CDs + *E. coli* probe in the absence and in the presence of *E. coli* sequence and the presence of non-complementary sequences (200 nM) or at different combination of them (200 nM *Salmonella* + 200 nM *Listeria* and 200 nM *Salmonella* + 200 nM *Listeria*)

To better understand the interaction of NR-CDs and *E. coli* DNA, UV–Vis and fluorescence titration experiments like those described above for calf thymus were now carried out keeping constant the NR-CDs concentration (2.8 µg/mL) and varying the concentration of ss or ds DNA sequences from *E. coli* (*E. coli* probe and *E. coli*/*E. coli* probe) instead calf thymus DNA. Results are shown in Fig. 4. UV–Vis titration shows the absorbance of NR-CDs initially increases until the ss *E. coli* concentration added reaches 100 nM (Fig. 4A) or 10 nM in the case of ds *E. coli* (Fig. 5B). Then, the increase of *E. coli* DNA concentration causes a decrease of absorbance, the same effect observed with calf thymus DNA. This result seems to indicate that the different behavior initially observed is due to the concentration rather than to the different DNA employed. This effect has been previously described for NR [45].

In the case of fluorescence titration, when ss *E. coli* concentration increases the fluorescence emission, intensity initially increases (Fig. 4C) at least until a concentration of 100 nM, followed by a slight fluorescence decrease for higher ss *E. coli* concentrations. In the case of ds *E. coli* (Fig. 4D), an increase in concentration always generates an increase of fluorescence at least in the studied range (10–2000 nM). This different behavior is the key point of the developed fluorescence assay.

At the optimal experimental conditions, the assay for *E. coli* detection was carried out using different concentrations of the target *E. coli* sequence (Fig. 5A). The fluorescence intensity of the solution containing only NR-CDs slightly increases when *E. coli* probe sequence is added. After addition of increasing amounts of the target sequence *E. coli*, and the hybridization event takes places, the fluorescence intensity increases proportionally to the target sequence concentration. Despite of the initial slight fluorescence intensity increase generates when NR-CDs interact with the ss *E. coli* probe sequence, the addition of other non-complementary sequence do not increase the fluorescence intensity as is demonstrated by the fluorescence titration of ss *E. coli* probe (Fig. 4C). This effect clearly demonstrates the fact that the emission intensity increases due to the formation of the corresponding dsDNA after hybridization of the target sequence with the probe, not to the addition of a nonspecific DNA sequence, where the hybridization event does not take place. The limit of detection for this assay was determined as 12 nM *E. coli* sequence (according to the 3 × *Sb* × m^−1^ criteria, where *Sb* corresponds to the standard deviation (*n* = 10) of *I*_0_ and m is the slope of the calibration plot), and the linear range is between 55 and 200 nM.

One of the most important aspects to take into account for analytical application is the study of the effect of potential interfering agents. For this reason, the selectivity of the fluorescence assay to detect specific *E. coli* sequences in the presence of other pathogens was carried out. We have studied the variation of the fluorescence signal of CDs-NR in samples containing other pathogen sequences (like *Salmonella* and *Listeria*) in the absence and in the presence of *Escherichia coli* sequences (Fig. 5C). It can be observed that fluorescence intensity increases from 290 to 403 a.u when *E. coli* sequence is present, whether or not is present in the DNA sequence of other bacteria. Furthermore, the presence of other non-complementary sequence modified the control experiment fluorescence intensity (probe + NR-CDs) in less than a 5%. From these results, it can be concluded that it is possible to detect a target sequence of *E. coli* in the presence of potential interfering sequences from other pathogen that usually can be present in samples containing *E. coli* bacteria.

### Real samples application

**Fig. 6:**
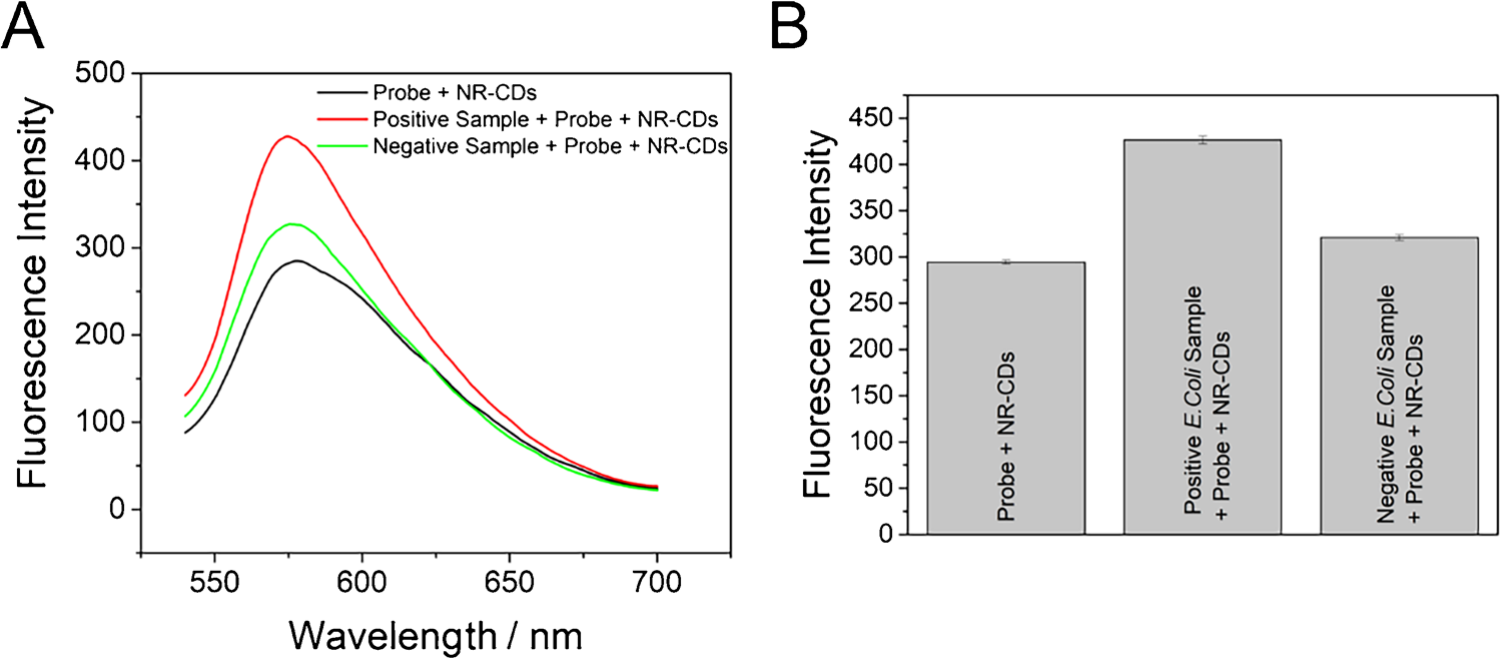
**A** Fluorescence spectra (*λ*_ex_ 530 nm) of NR-CDs (2.8 µg/mL) with the *E. coli* probe before (black) and after the incubation with two bacteria samples: sample containing *E. coli* (red), sample containing *Lactobacillus rhamnosus* (green). **B** Fluorescence intensity (*λ*_em_ 580 nm) obtained from spectra in **A**

The next step in this work was to apply the developed strategy to real *E. coli* samples. In particular, two samples, obtained by bacteria culture (see “Materials and methods” section), were analyzed using the developed fluorescence assay. One of them has been confirmed positive on presence of *E. coli*, while the other one has been confirmed negative in the presence of this bacteria, but another microorganism (*Lactobacillus rhamnosus*) has been grown in it. Both samples were analyzed applying the developed assay, and the obtained results clearly showed a great difference between both samples (Fig. 6). As can be observed, compared to the control (probe + NR-CDs), the fluorescence increase in the case of the negative sample is around (9 ± 1) %, while in the positive sample, the fluorescence increase is of (45 ± 1) %. The big difference between two samples and with the control demonstrates the applicability of the developed fluorescence assay and stablishing a great tool for qualitative identification of bacteria type.

The analytical parameters of our assay have been compared with similar assays using carbon dots as fluorescence probes to detect DNA hybridization (see Table S1). Despite most of these works present better limit of detection and linear range, the developed assay has some advantages as the easy synthesis of NR-CD in one step and the possibility of exciting in the visible region which avoid potential interferents and create a very selective platform. Furthermore, while other authors only analyze synthetic samples, we have also tested the applicability of the developed assay by analyzing real samples of culture bacteria.

## Conclusions

Carbon nanodots modified with Neutral Red (NR-CDs) have been synthesized including the phenazine colorant (NR) in the synthesis process, achieving the covalent linkage of NR molecules in the nanomaterial structure. The formation of the NR modified nanomaterial has been assessed by different techniques, confirming the generation of covalent bonds between the phenazine molecules and the CDs structure. Moreover, following the changes in the UV–Visible absorption and fluorescence of the NR modified nanomaterial, its interaction with DNA has been studied. It is demonstrated that NR-CDs interact with DNA, and they have a different interaction way with singled or doubled stranded DNA. These studies have been successfully applied to develop a fluorescence assay for the detection of specific DNA sequences of *Escherichia coli*. When the hybridization between the probe and the target sequences happens, the interaction of the dsDNA formed with NR-CDs gives rise to an increase of the fluorescence emission associated with the stabilization of the protonated form of the phenazine molecule inserted in the nanomaterial. The assay has been successfully applied to detect *E. coli* in bacteria culture samples, by the difference in fluorescence intensity between samples containing or not *E. coli* bacteria. A great advantage of the developed assay is the use of an excitation wavelength in the visible region, simplifying the instrumentation requires to be carried out.

## Supplementary Information

Below is the link to the electronic supplementary material.

Supplementary file1 (DOCX 541 KB)

## References

[1] Maiti D, Tong X, Mou X, Yang K. Carbon-based nanomaterials for biomedical applications: a recent study. Front Pharmacol. 2019;9:1401. 10.3389/fphar.2018.01401. 10.3389/fphar.2018.01401 PMC6421398 30914959

[2] Sengupta J. Handbook of nanomaterials for manufacturing applications. Chapter 17 - application of carbon nanomaterials in the electronic industry. 2020. pp. 421–50. 10.1016/B978-0-12-821381-0.00017-X.

[3] Mauter MS, Elimelech M (2008). Environmental applications of carbon-based nanomaterials. Environ Sci Technol.

[4] Banerjee J, Dutta K, Rana D, Rajendran S, Naushad M, Raju K, Boukherroub R (2019). Carbon nanomaterials in renewable energy production and storage applications. Emerging nanostructured materials for energy and environmental science.

[5] Baptista FR, Belhout SA, Giordani S, Quinn SJ (2015). Recent developments in carbon nanomaterial sensors. Chem Soc Rev.

[6] Ray SC, Jana NR. Carbon nanomaterials for biological and medical applications. Chapter 3 - application of carbon-based nanomaterials as biosensor. 2017. pp. 87–127. 10.1016/B978-0-323-47906-6.00003-5.

[7] Georgakilas V, Otyepka M, Bourlinos AB, Chandra V, Kim N, Kemp KC (2012). Functionalization of graphene: covalent and non-covalent approaches, derivatives and applications. Chem Rev.

[8] Lu F, Gu L, Meziani MJ, Wang X, Luo PG, Veca LM (2009). Advances in bioapplications of carbon nanotubes. Adv Mater.

[9] Mateo-Alonso A, Bonifazi D, Prato M, Dai L (2006). Chapter 7—functionalization and applications of [60]fullerene. Carbon nanotechnology.

[10] Ðorđević L, Arcudi F, Prato M (2019). Preparation, functionalization and characterization of engineered carbon nanodots. Nat Protoc.

[11] Jolly A, Miao D, Daigle M, Morin J-F (2020). Emerging bottom-up strategies for the synthesis of graphene nanoribbons and related structures. Angew Chem Int Ed.

[12] Wu K, Cao X, Li M, Lei B, Zhan J, Wu M (2020). Bottom-up synthesis of MoS2/CNTs hollow polyhedron with 1T/2H hybrid phase for superior potassium-ion storage. Small.

[13] Xu X, Ray R, Gu Y, Ploehn HJ, Gearheart L, Raker K (2004). Electrophoretic analysis and purification of fluorescent single-walled carbon nanotube fragments. J Am Chem Soc.

[14] Xiao L, Sun H (2018). Novel properties and applications of carbon nanodots. Nanoscale Horiz.

[15] Arcudi F, Đorđević L, Prato M (2016). Synthesis, separation, and characterization of small and highly fluorescent nitrogen-doped carbon nanodots. Angew Chem Int Ed.

[16] Liu H, Zhang Y, Huang C (2019). Development of nitrogen and sulfur-doped carbon dots for cellular imaging. J Pharm Anal.

[17] Xu Q, Li B, Ye Y, Cai W, Li W, Yang C (2018). Synthesis, mechanical investigation, and application of nitrogen and phosphorus co-doped carbon dots with a high photoluminescent quantum yield. Nano Res.

[18] Rigodanza F, Đorđević L, Arcudi F, Prato M (2018). Customizing the electrochemical properties of carbon nanodots by using quinones in bottom-up synthesis. Angew Chem Int Ed.

[19] Li Y, Zheng X, Zhang X, Liu S, Pei Q, Zheng M (2017). Porphyrin-based carbon dots for photodynamic therapy of hepatoma. Adv Healthc Mater.

[20] Hadla M, Halabi MA. Comprehensive analytical chemistry. Chapter three - effect of quorum sensing, volume 81. 2018. pp. 95–116. 10.1016/bs.coac.2018.02.004.

[21] Pauliukaite R, Ghica ME, Barsan MM, Brett CMA (2010). Phenazines and polyphenazines in electrochemical sensors and biosensors. Anal Lett.

[22] Barsan MM, Pinto EM, Brett CMA (2008). Electrosynthesis and electrochemical characterisation of phenazine polymers for application in biosensors. Electrochim Acta.

[23] Xiao-Ni Q, Dang L-R, Qu W-J, Zhang Y-M, Yao H, Lin Q (2020). Phenazine derivatives for optical sensing: a review. J Mater Chem C.

[24] Zozulya V, Blagoi Y, Löber G, Voloshin I, Winter S, Makitruk V (1997). Fluorescence and binding properties of phenazine derivatives in complexes with polynucleotides of various base compositions and secondary structures. Biophys Chem.

[25] Naha S, Velmathi S (2019). Phenazine-based fluorescence “turn-off” sensor for fluoride: application on real samples and to cell and zebrafish imaging. ChemistrySelect.

[26] Lu X, Lan T, Qin Z, Wang Z-S, Zhou G (2014). A Near-infrared dithieno[2,3-a:3′,2′-c]phenazine-based organic co-sensitizer for highly efficient and stable quasi-solid-state dye-sensitized solar cells. ACS Appl Mater Interfaces.

[27] Shi J, Chen J, Chai Z, Wang H, Tang R, Fan K (2012). High performance organic sensitizers based on 11,12-bis(hexyloxy) dibenzo[a, c]phenazine for dye-sensitized solar cells. J Mater Chem.

[28] Richard CA, Pan Z, Hsu H-Y, Cekli S, Schanze KS, Reynolds JR (2014). Effect of isomerism and chain length on electronic structure, photophysics, and sensitizer efficiency in quadrupolar (donor)2–acceptor systems for application in dye-sensitized solar cells. ACS Appl Mater Interfaces.

[29] Hu X, Shi J, Shi Y, Zou X, Tahir HE, Holmes M (2019). A dual-mode sensor for colorimetric and fluorescent detection of nitrite in hams based on carbon dots-neutral red system. Meat Sci.

[30] Yang W, Ni J, Luo F, Weng W, Wei Q, Lin Z (2017). Cationic carbon dots for modification-free detection of hyaluronidase via an electrostatic-controlled ratiometric fluorescence assay. Anal Chem.

[31] Azizi S, Gholivand M-B, Amiri M, Manouchehri I (2020). DNA biosensor based on surface modification of ITO by physical vapor deposition of gold and carbon quantum dots modified with neutral red as an electrochemical redox probe. Microchem J.

[32] Li L, Shi L, Jia J, Eltayeb O, Lu W, Tang Y (2021). Red fluorescent carbon dots for tetracycline antibiotics and pH discrimination from aggregation-induced emission mechanism. Sensors Actuators B Chem.

[33] Dan C, Zhao Z, Feng J, Xin Y, Yang Y, Shi L (2021). Lysosome-targeted red-fluorescent carbon dots for turn-on detection of permanganate and pH in vivo and in vitro. Sensors Actuators B Chem.

[34] Gao W, Song H, Wang X, Liu X, Pang X, Zhou Y (2018). Carbon dots with red emission for sensing of Pt2+, Au3+, and Pd2+ and their bioapplications in vitro and in vivo. ACS Appl Mater Interfaces.

[35] Law JW, Ab Mutalib NS, Chan KG, Lee LH. Rapid methods for the detection of foodborne bacterial pathogens: principles, applications, advantages and limitations. Front Microbiol. 2015;5:770. 10.3389/fmicb.2014.00770. 10.3389/fmicb.2014.00770 PMC4290631 25628612

[36] Su D, Li H, Yan X, Lin Y, Lu G (2021). Biosensors based on fluorescence carbon nanomaterials for detection of pesticides. TrAC Trends Anal Chem.

[37] García-Mendiola T, Bravo I, López-Moreno JM, Pariente F, Wannemacher R, Weber K (2018). Carbon nanodots based biosensors for gene mutation detection. Sensors Actuators B Chem.

[38] Guerrero-Esteban T, Gutiérrez-Sánchez C, Martínez-Periñán E, Revenga-Parra M, Pariente F, Lorenzo E (2021). Sensitive glyphosate electrochemiluminescence immunosensor based on electrografted carbon nanodots. Sensors Actuators B Chem.

[39] Meehan T, Gamper H, Becker JF (1982). Characterization of reversible, physical binding of benzo[a]pyrene derivatives to DNA. J Biol Chem.

[40] Mostazo-López MJ, Ruiz-Rosas R, Tagaya T, Hatakeyama Y, Shiraishi S, Morallón E (2020). Nitrogen doped superactivated carbons prepared at mild conditions as electrodes for supercapacitors in organic electrolyte. C—J Carbon Res.

[41] Liu D, Lei W, Portehault D, Qin S, Chen Y (2015). High N-content holey few-layered graphene electrocatalysts: scalable solvent-less production. J Mater Chem A.

[42] Susi T, Pichler T, Ayala P (2015). X-ray photoelectron spectroscopy of graphitic carbon nanomaterials doped with heteroatoms. Beilstein J Nanotechnol.

[43] Breslin DT, Yu C, Ly D, Schuster GB (1997). Structural modification changes the DNA binding mode of cation-substituted anthraquinone photonucleases: association by intercalation or minor groove binding determines the DNA cleavage efficiency. Biochemistry.

[44] Pyle AM, Rehmann JP, Meshoyrer R, Kumar CV, Turro NJ, Barton JK (1989). Mixed-ligand complexes of ruthenium(II): factors governing binding to DNA. J Am Chem Soc.

[45] Jiang X, Shang L, Wang Z, Dong S (2005). Spectrometric and voltammetric investigation of interaction of neutral red with calf thymus DNA: pH effect. Biophys Chem.

