# Supplementary material for: Neutral Red-carbon nanodots for selective fluorescent DNA sensing
Source: Anal Bioanal Chem. 2022 Mar 14;414(18):5537–48. doi: 10.1007/s00216-022-03980-1 (PMC9242914; doi:10.1007/s00216-022-03980-1)
Supplement: Supplementary file 1 — Supplementary file1 (DOCX 541 KB) [file 216_2022_3980_MOESM1_ESM.docx]

***Supplementary Information (SI)***

**Neutral Red-carbon nanodots for selective fluorescent DNA sensing.**

Emiliano Martínez-Periñán* ^a^, Álvaro Martínez-Sobrino ^a^, Iria Bravo ^a^, Tania García-Mendiola ^a,b^, Eva Mateo-Martí ^c^, Félix Pariente ^a,d^, Encarnación Lorenzo* ^a,b,d^.

^a^ Departamento de Química Analítica y Análisis Instrumental, Universidad Autónoma de Madrid, 28049, Madrid, Spain.

^b^ Institute for Advanced Research in Chemical Sciences (IAdChem), Universidad Autónoma de Madrid, 28049, Madrid, Spain.

^c^ Centro de Astrobiología (CSIC-INTA), Ctra. Ajalvir, Km. 4, 28850, Torrejón de Ardoz, Madrid, Spain.

^d^ IMDEA-Nanociencia, Ciudad Universitaria de Cantoblanco, 28049, Madrid, Spain.

∗Corresponding author at: [emiliano.martinez@uam.es](mailto:emiliano.martinez@uam.es) and [encarnacion.lorenzo@uam.es](mailto:encarnacion.lorenzo@uam.es)


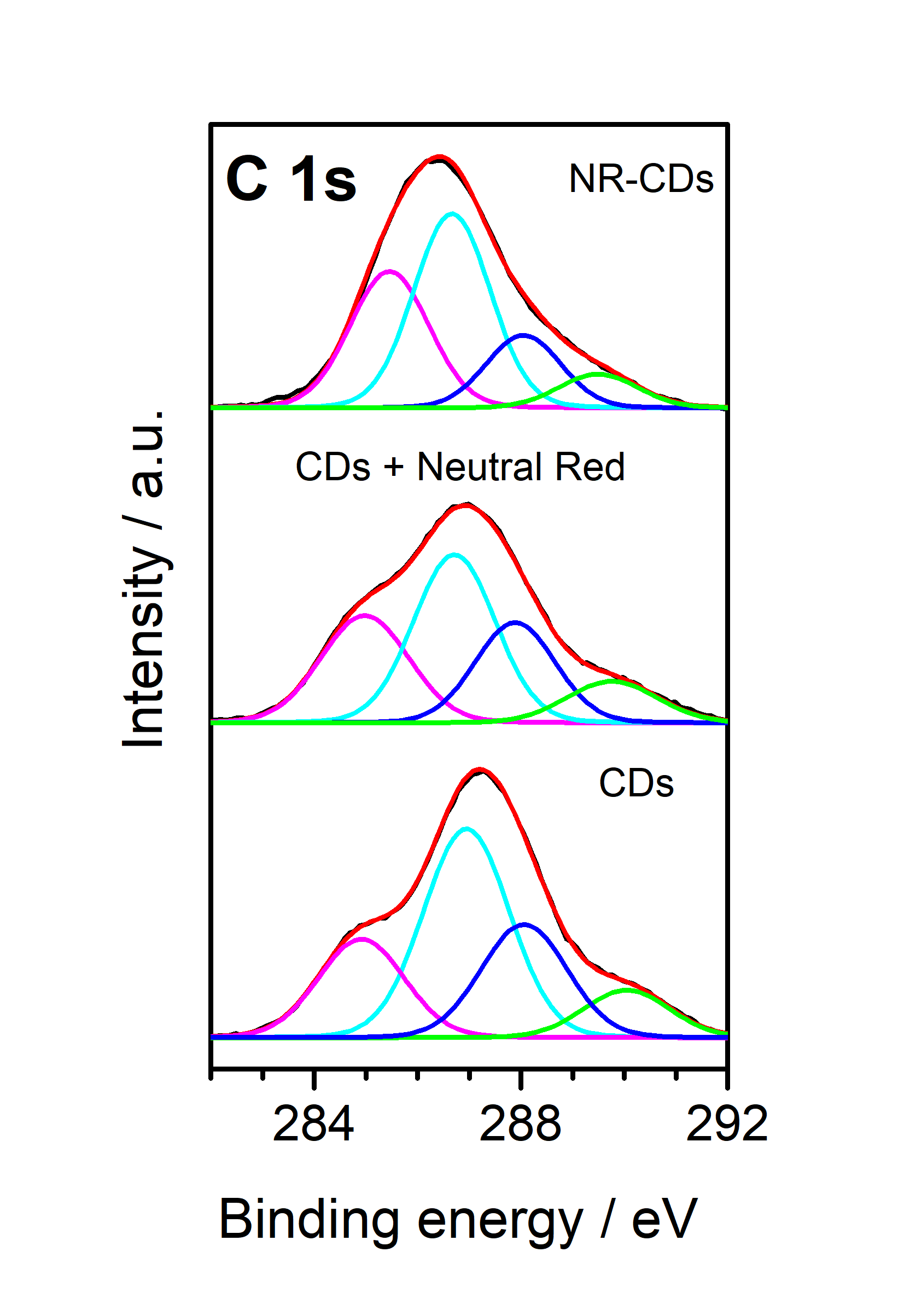


Figure SI 1. XPS spectra of C 1s core level for the gold surfaces of: NR-CDs, CDs + NR and CDs.


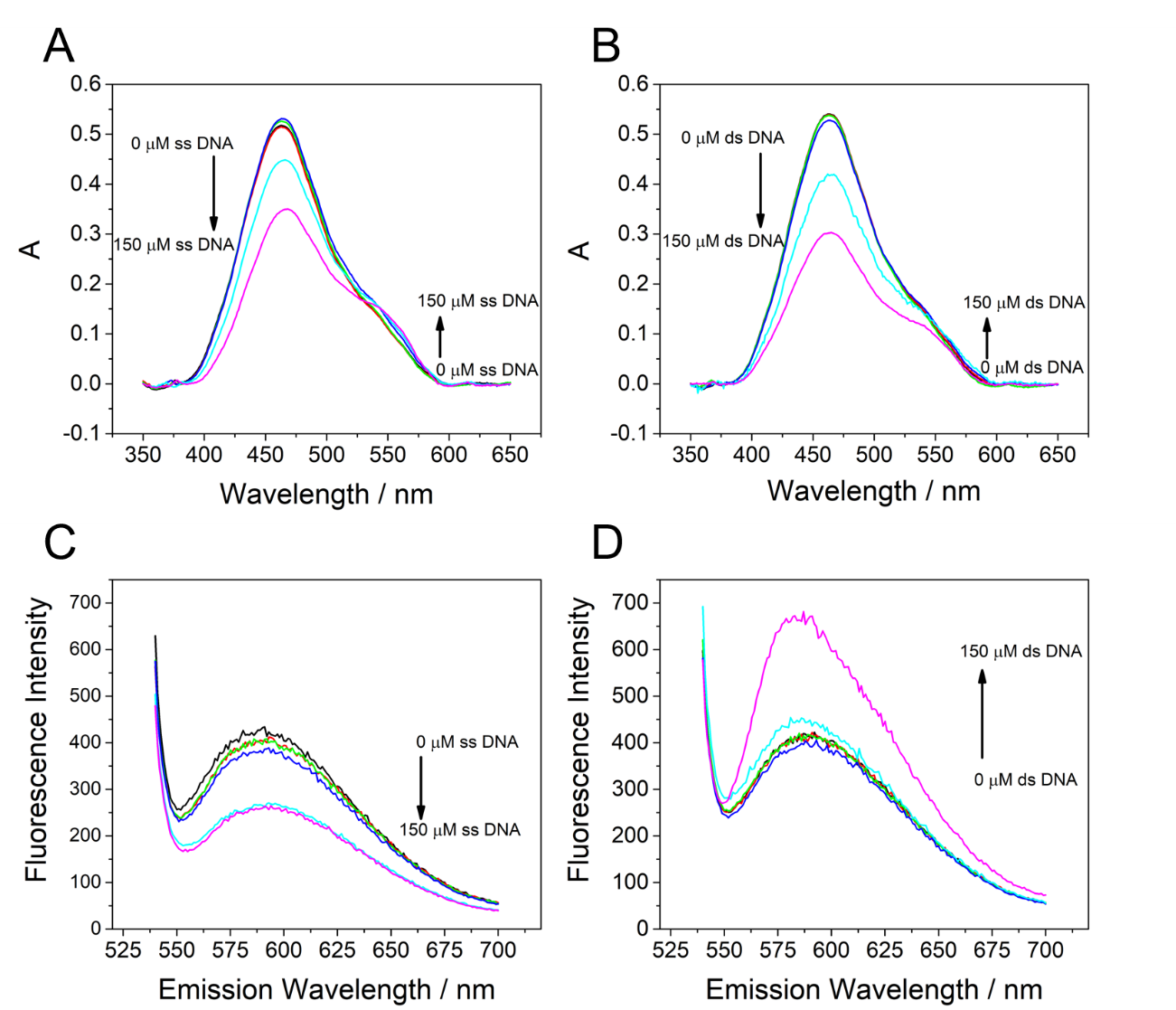


Figure SI 2. UV-Vis absorption spectra (A, B) or fluorescence spectra (C, D) of NR-CDs at 0.1 M PB pH 7 in the absence and in the presence of increasing concentrations (0- 150 µM) of ssDNA (A, C) and dsDNA (B,D).


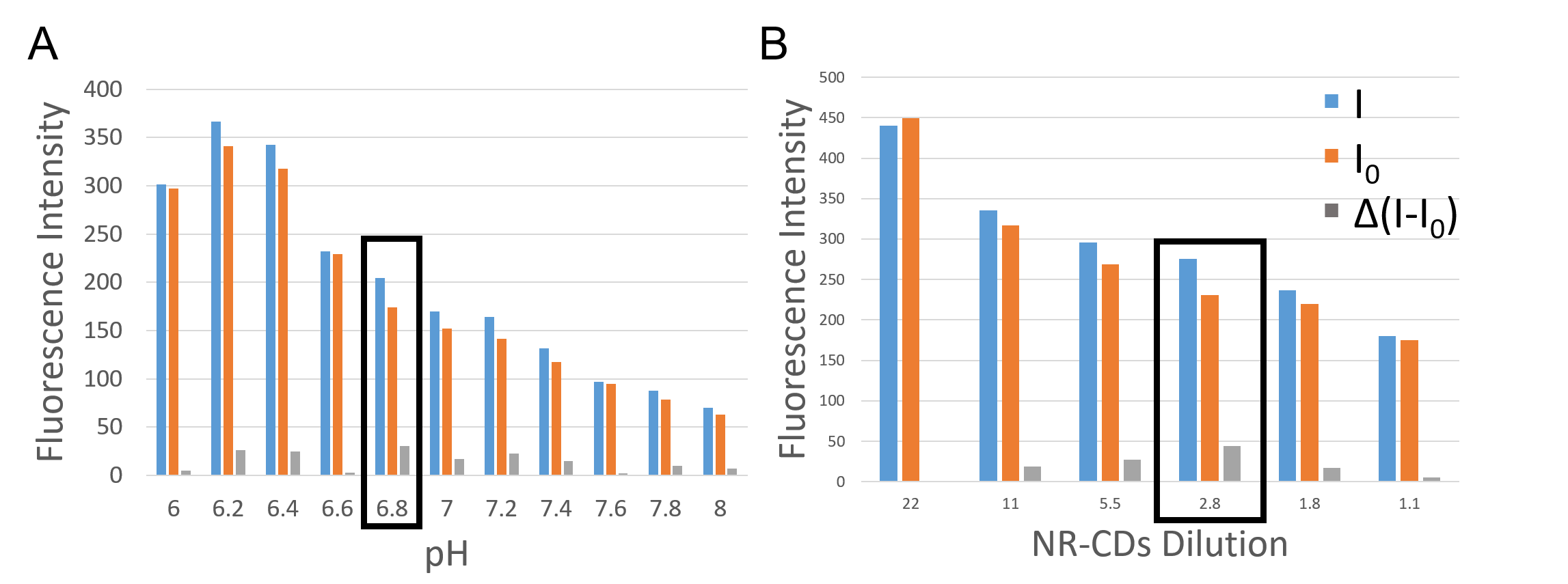


Figure SI 3. (A) Fluorescence emission of NR-CDs (2.8 µg/mL) with *E. Coli* Probe (200 nM) before (I_0_) and after (I) the incubation with *E. Coli complementary* sequence (200 nM) at different pHs 0.1 M PB buffer. (B) Fluorescence emission of NR-CDs at different concentrations with *E. Coli* Probe (200 nM) before (I_0_) and after (I) the incubation with *E. Coli complementary* sequence (200 nM) in 0.1 M PB buffer pH 6.8. Δ(I-I_0_) represent the difference in emission of I-I_0_.

Table S1.Analytical parameter of different work published using carbon dots as fluorescence probe for DNA hybridization detection.

| **Nanomaterial** | **LOD** | **Linear range** | **λ_ex_ / nm** | **λ_em_ / nm** | **Reference** |
| --- | --- | --- | --- | --- | --- |
| Carbon dots covalently attached to the 5′ phosphate groups of various monobase | 9.9 pM | 10 pM–0.5 μΜ | 400 | 470 | (1) |
| Carbon dots | No data | No data | 375 | 445 | (2) |
| Carbon Dots | 270 pM | Up to 200 nM | 340 | 440 | (3) |
| Thiazole Orange-Modified Carbon Dots (supramolecular structures) | 0.9 nM | No data | 370 | 450 | (4) |
| NR-CDs | 12 nM | 12- 200 nM | 530 | 580 | This work |

**Bibliography:**

1. Motaghi H, Mehrgardi MA. Spectrofluorometric genotyping of single nucleotide polymorphisms using carbon dots as fluorophores. Spectrochimica Acta Part A: Molecular and Biomolecular Spectroscopy. 2019;206:154-9.

2. Liang C-Y, Xia W, Yang C-Z, Liu Y-C, Bai A-M, Hu Y-J. Exploring the binding of carbon dots to calf thymus DNA: From green synthesis to fluorescent molecular probe. Carbon. 2018;130:257-66.

3. García-Mendiola T, Elosegui CG, Bravo I, Pariente F, Jacobo-Martin A, Navio C, et al. Fluorescent C-NanoDots for rapid detection of BRCA1, CFTR and MRP3 gene mutations. Microchimica Acta. 2019;186(5):293.

4. Jin M, Liu X, Zhang X, Wang L, Bing T, Zhang N, et al. Thiazole Orange-Modified Carbon Dots for Ratiometric Fluorescence Detection of G-Quadruplex and Double-Stranded DNA. ACS Applied Materials & Interfaces. 2018;10(30):25166-73.
